# Towards a strategy for malaria in pregnancy in Afghanistan: analysis of clinical realities and women’s perceptions of malaria and anaemia

**DOI:** 10.1186/s12936-015-0964-0

**Published:** 2015-11-04

**Authors:** Natasha Howard, Sayed Enayatullah, Nader Mohammad, Ismail Mayan, Zohra Shamszai, Mark Rowland, Toby Leslie

**Affiliations:** London School of Hygiene and Tropical Medicine (LSHTM), London, UK; HealthNet-TPO (HNTPO), Kabul, Afghanistan; Health Protection and Research Organization (HPRO), Kabul, Afghanistan; Care International, Kabul, Afghanistan

**Keywords:** Maternal malaria, Anaemia, Low birthweight, *Plasmodium vivax*, IPTp

## Abstract

**Background:**

Afghanistan has some of the worst maternal and infant mortality indicators in the world and malaria is a significant public health concern. Study objectives were to assess prevalence of malaria and anaemia, related knowledge and practices, and malaria prevention barriers among pregnant women in eastern Afghanistan.

**Methods:**

Three studies were conducted: (1) a clinical survey of maternal malaria, maternal anaemia, and neonatal birthweight in a rural district hospital delivery-ward; (2) a case–control study of malaria risk among reproductive-age women attending primary-level clinics; and (3) community surveys of malaria and anaemia prevalence, socioeconomic status, malaria knowledge and reported behaviour among pregnant women.

**Results:**

Among 517 delivery-ward participants (1), one malaria case (prevalence 1.9/1000), 179 anaemia cases (prevalence 346/1000), and 59 low-birthweight deliveries (prevalence 107/1000) were detected. Anaemia was not associated with age, gravidity, intestinal parasite prevalence, or low-birthweight at delivery. Among 141 malaria cases and 1010 controls (2), no association was found between malaria infection and pregnancy (AOR 0.89; 95 % CI 0.57–1.39), parity (AOR 0.95; 95 % CI 0.85–1.05), age (AOR 1.02; 95 % CI 1.00–1.04), or anaemia (AOR 1.00; 95 % CI 0.65–1.54). Those reporting insecticide-treated net usage had 40 % reduced odds of malaria infection (AOR 0.60; 95 % CI 0.40–0.91). Among 530 community survey participants (3), malaria and anaemia prevalence were 3.9/1000 and 277/1000 respectively, with 34/1000 experiencing severe anaemia. Despite most women having no formal education, malaria knowledge was high. Most expressed reluctance to take malaria preventive medication during pregnancy, deeming it potentially unsafe.

**Conclusions:**

Given the low malaria risk and reported avoidance of medication during pregnancy, intermittent preventive treatment is hard to justify or implement. Preventive strategy should instead focus on long-lasting insecticidal nets for all pregnant women.

## Background

Infection with *Plasmodium falciparum* or *Plasmodium vivax* can be dangerous in pregnancy, increasing risks of severe anaemia, premature delivery, low-birthweight, and foetal, neonatal and maternal death [1, 2]. Falciparum infection in pregnancy causes up to approximately 10,000 maternal deaths, 3–8 % infant deaths, and 8–14 % low-birthweight (LBW) deliveries annually, and pregnant women are often prioritized for preventive interventions including long-lasting insecticidal nets (LLINs) and intermittent preventive treatment (IPT) [3, 4]. In unstable transmission settings, such as much of South Asia, pregnant women experience 2–3× higher risk than non-pregnant women of developing severe malaria or malaria-related severe anaemia [2, 4–6].

Malaria in pregnancy is most studied for falciparum infection in sub-Saharan Africa and Southeast Asia [3, 7, 8]. Though less documented, growing literature on vivax infection in pregnancy indicates considerable risk and approximately 71 million pregnancies in vivax-endemic areas globally [1, 4, 6, 9–15]. *Plasmodium vivax* is the most geographically widespread human malaria parasite—endemic in much of Asia, including Afghanistan, with approximately 2.9 billion people at risk globally [15–19]. It has been associated with severe clinical manifestations in pregnancy, including severe anaemia, thrombocytopenia, miscarriage, low-birthweight, and preterm delivery [1, 4, 19, 20]. Though in vivo *P. vivax* placental sequestration has not been identified, in vitro evidence exists of cytoadherence to placental glycosaminoglycans [21]. Relapsing infections from hypnozoite reservoirs cannot be treated effectively, as primaquine is contraindicated in pregnancy, making prevention preferable [22].

Afghanistan has received considerable international attention and support since the Taliban government ended in 2001, but remains a fragile country [23, 24]. The estimated maternal mortality ratio reduced from 1300/100,000 in 2000 to 460/100,000 in 2010 [25]. Similarly, the infant mortality rate reduced from 153/1000 to 74/1000 in 2011 [26, 27]. However, indicators remain poor. An estimated 16 million people are at risk of malaria in Afghanistan and it remains a public health concern [28, 29]. Transmission is unstable and seasonal, and a significant cause of morbidity in lowland and rice irrigation areas [29].

No publications were found on malaria epidemiology in pregnancy in Afghanistan. This study aimed to assess the contribution of malaria to maternal anaemia and birth outcomes in eastern Afghanistan. Objectives were to assess: (1) prevalence of malaria and anaemia among pregnant women, (2) risks of malaria in pregnancy, and (3) malaria awareness and reported behaviours during pregnancy.

## Methods

### Study site and population

**Fig. 1:**
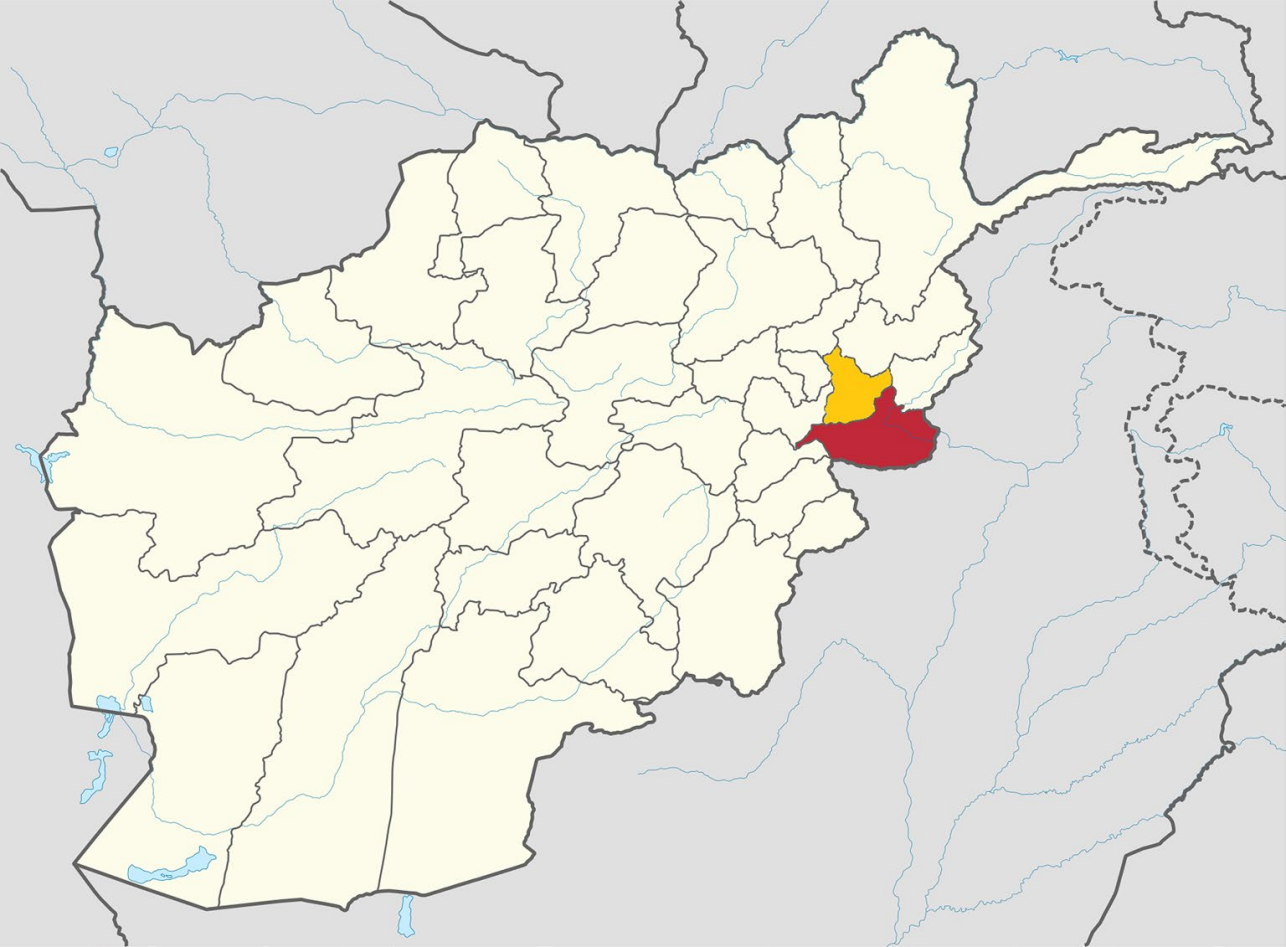
Map of Afghanistan showing Nangarhar and Laghman Provinces. Adapted from Tubbs, https://en.wikipedia.org/wiki/Nangarhar_Province#/media/File:Nangarhar_in_Afghanistan.svg

Studies were conducted in Ghani Khel District Hospital, a secondary-level facility in rural Nangarhar province, and primary-level facilities and communities within its catchment area (Fig. 1). The target population was women of reproductive age (WRA; aged 15–49) from among an estimated population of 500,000 women accessing health services subcontracted to an NGO provider, HealthNet-TPO, through Afghanistan’s Basic Package of Health Services [30]. Malaria transmission is moderate and seasonal, vivax peaking May–August and causing 70–95 % of malaria, and falciparum predominantly in October–November [31]. Main vectors are *Anopheles stephensi*, *Anopheles superpictus*, and *Anopheles culicifacies* [29, 31]. At the time of this study, insecticide-treated nets (e.g. ITNs/LLINs)—targeted preferentially to pregnant women—were distributed at subsidized prices through primary-level facilities [32, 33].

### Study design and data collection

Three complementary studies were conducted: (1) a clinical survey of maternal malaria, maternal anaemia, and neonatal birthweight in the district hospital (2) a case–control study of malaria risk factors among WRA attending nearby primary-level facilities, and (3) a two-round community survey of malaria and anaemia prevalence, socioeconomic status and related knowledge, attitudes and practices among pregnant women in four nearby districts.

#### Delivery-ward survey

Women and chaperones were informed and asked about participation during ward admission, and those giving informed consent were enrolled and examined between February and December 2004: (1) thick and thin Giemsa-stained blood smears of peripheral, cord and placental blood were tested for malaria; (2) maternal blood tested for haemoglobin; (3) stool tested for intestinal parasites; and (4) neonate and placenta weighed at delivery. Blood slides were collected by a trained midwife, stained and examined the same day by a trained microscopist at 100× magnification, with 200 fields checked before recording a negative result. All slides were re-read by an experienced microscopist, blinded to original diagnosis. Discrepancies were resolved by a third blinded reading. Participants found positive for malaria were treated according to national guidelines (i.e. from 2nd trimester, CQ 25 mg/kg × 3 days for vivax or SP 25 mg/kg × 1 day plus artesunate 4 mg/kg × 3 days for falciparum malaria). Haemoglobin was measured by attending midwife using a HemoCue point-of-care test (Ängelholm, Sweden). Participants found anaemic were provided treatment according to national guidelines (i.e. iron 120 mg plus folic acid 0.8 mg daily for 3 months). Stool was examined for helminths and intestinal protozoa by trained microscopists, using duplicate Kato-Katz thick smears prepared shortly after collection and allowed to clear for 45–60 min before examination [34]. Participants found positive were treated according to national guidelines (i.e. from 2nd trimester, mebendazole 500 mg). Birthweight was measured by attending midwife for all births within 1 h of delivery, using an electronic scale (Salter, Birmingham UK) accurate to ±10 g and calibrated weekly [35]. Fresh placentas were weighed by attending midwife, untrimmed and without blood expressed, using the same procedure [36]. Data were double-entered into Microsoft^®^ Access 2000, with range and consistency checks to reduce transposition errors.

#### Case–control study

All reproductive-age female outpatients presenting with suspected malaria (i.e. fever or history of fever suspected to be malaria) and providing informed consent, were enrolled between September 2004 and January 2005 at basic health facilities with malaria diagnostics and staff willing to participate in a study. All patients were examined by clinic doctors and clinical diagnosis, age, marital status, number of children, and number of previous pregnancies recorded on standardized forms. Controls were defined as WRA with clinically-suspected malaria, negative blood slides and no recent history of malaria (i.e. to exclude recently-treated patients). Cases were defined as WRA with microscopically confirmed malaria and categorized according to malaria species and severity. Severe cases were defined as parasitaemic, with one or more WHO indicators for severe malaria and absence of identified alternative causes [37]. Microscopy, treatment, and data entry replicated delivery survey protocols.

#### Community survey

Multi-stage sampling was used: (1) four districts were selected with functioning community health infrastructure (i.e. Shinwar, Mohmand Dara, Batikot, Nazian); (2) villages were identified within each district that were malaria-endemic, with antenatal services, and accessible by four-wheel drive vehicle; (3) all pregnant women in each village were invited to participate. Sample size was calculated to detect a malaria prevalence of 5 % with 80 % power and 95 % confidence. Two collection periods, May–June and December 2004, incorporated seasonal peaks of vivax and falciparum transmission respectively. Those providing informed consent were tested for malaria and anaemia and answered a structured questionnaire. Adapted from previous research [32], the questionnaire was back-translated in Pashtu and piloted in a non-participating district. Literate female interviewers were recruited from local communities and trained over 3 days on privacy, prompting, and questionnaire completion. Microscopy, treatment, and data entry replicated delivery survey protocols.

### Analysis

Data was analysed using Stata/IC13.1.

#### Delivery-ward survey

Malaria infection was categorized as negative (i.e. negative peripheral, umbilical, and placental slide result) or positive (i.e. any positive result). Anaemia was categorized as non-anaemic (i.e. 110 g/l or above) or anaemic (i.e. below 110 g/l) [38]. Birthweight was categorized as non-LBW (i.e. 2500 g or above) or LBW (i.e. below 2500 g). Intestinal parasites were categorized as absent (i.e. none detected) or present (i.e. detection of any helminth or protozoa). Logistic regression was used to calculate odds ratios of anaemia or LBW outcomes, with univariate regression providing crude estimates and multivariate regression adjusting for a priori confounders (i.e. age, gravidity, presence of intestinal parasites). Cell sizes below 30 prompted exact logistic methods. Effect modifiers (i.e. significant LRT test) were reported individually.

#### Case–control study

Categorization and analysis replicated delivery survey protocols. Additionally, ITN usage was defined as reporting sleeping under ITNs the previous night. Logistic regression calculated odds ratios of exposures. A priori confounders were district, facility, age, and parity.

#### Community survey

Categorization and analysis replicated delivery survey protocols. Additionally, participant age, education, housing, and household asset variables were weighted and scored within a socioeconomic status (SES) index using principal components analysis [32, 39]. Logistic regression calculated odds ratios of anaemia. A priori confounders were SES, district, age, parity, trimester, and season.

### Ethics

Approval was provided by the Ministry of Public Health in Afghanistan and the Research Ethics Committee of the London School of Hygiene and Tropical Medicine in the UK (reference 5508). All participants were informed about the study purpose, content and potential publication and written or verbal informed consent was recorded. Data was coded anonymously and stored in password-protected hard-drives.

## Results

### Delivery-ward survey

In total, 517 patients agreed to participate. Average age was 25 years (range 16–40). Approximately 35 % were primigravida, 31 % multigravida (2–4 pregnancies) and 33 % grand-multigravida (5+ pregnancies). Among 38 women with intestinal parasites (prevalence 74/1000, 95 % CI 54–99), most frequent were *Entamoeba histolytica* (47 %), *Giardia lamblia* (26 %) and *Ascaris lumbricoides* (16 %). One malaria case (prevalence 1.9/1000, 95 % CI 0.3–13.7), 179 anaemia cases (prevalence 346/1000, 95 % CI 306–388), and 59 low-birthweight (LBW) deliveries (114/1000, 95 % CI 89–115) were recorded.

As only one malaria case was detected, further analysis only compared factors associated with anaemia and LBW (Table 1). Anaemia presence was not associated with age, gravidity, presence of intestinal parasites, or LBW delivery. LBW delivery was not associated with intestinal parasite or anaemia presence, though it was associated with gravidity in multivariate analysis.

**Table 1:** Associations of demographic and clinical variables with maternal anaemia and low-birthweight delivery among 517 delivery-ward patients in eastern Afghanistan

Associations with anaemia	Anaemic, n (%)	Non-anaemic, n (%)	OR (95 % CI)	AOR (95 % CI)
	(N = 179)	(N = 338)		
Age group				
15–20	66 (36.9)	143 (42.3)	Ref.	Ref.
21–30	84 (46.9)	154 (45.6)	1.18 (0.80–1.75)	1.00 (0.63–1.60)
31–49	29 (16.2)	41 (12.1)	1.53 (0.88–2.68)	1.20 (0.59–2.44)
Gravidity				
Primigravidous	54 (30.2)	129 (38.2)	Ref.	Ref.
Multigravidous (2–4)	58 (32.4)	104 (30.8)	1.33 (0.85–2.09)	1.32 (0.81–2.17)
Grand-multigravidous (5+)	67 (37.4)	105 (31.1)	1.52 (0.98–2.37)	1.42 (0.80–2.53)
Intestinal parasites				
No	167 (93.3)	312 (92.3)	Ref.	Ref.
Yes	12 (6.7)	26 (7.7)	0.86 (0.42–1.75)	0.82 (0.40–1.67)
LBW				
No	159 (88.8)	229 (88.5)	Ref.	Ref.
Yes	20 (11.2)	39 (11.5)	0.96 (0.54–1.71)	1.09 (0.60–1.97)

Associations with LBW	LBW, n (%)	Non-LBW, n (%)	OR (95 % CI)	AOR (95 % CI)
	(N = 59)	(N = 458)		
Age				
15–20	33 (55.9)	176 (38.4)	Ref.	Ref.
21–30	21 (35.6)	217 (47.4)	0.52 (0.29–0.92)*	1.13 (0.57–2.22)
31–49	5 (8.5)	65 (14.2)	0.41 (0.15–1.10)	2.17 (0.55–8.49)
Gravidity				
Primigravidous	38 (64.4)	145 (31.7)	Ref.	Ref.
Multigravidous (2–4)	13 (22.0)	149 (32.5)	0.33 (0.17–0.64)**	0.31 (0.15–0.64)**
Grand-multigravidous (5+)	8 (13.6)	164 (35.8)	0.19 (0.08–0.41)**	0.13 (0.04–0.39)**
Intestinal parasites				
No	55 (93.2)	424 (92.6)	Ref.	Ref.
Yes	4 (6.8)	34 (7.4)	0.90 (0.31–2.65)	1.04 (0.34–3.14)
Anaemia				
No	39 (66.1)	299 (65.3)	Ref.	Ref.
Yes	20 (33.9)	159 (34.7)	0.96 (0.54–1.70)	1.09 (0.60–1.96)

* p < 0.05; ** p < 0.001; AOR adjusted for age, gravidity, intestinal parasite presence. Cell sizes below 30 use exact logistic methods

### Case–control study

In total, 141 malaria cases and 1010 controls were enrolled from reproductive-age women attending eight district clinics (Table 2). Most were resident in Jalalabad (40 %), Shinwar (23 %) and Momand Dara (15 %) districts. Average age was 28 (range 15–45). Most (81 %) were married. Approximately 25 % were nulliparous, 38 % had delivered 1–5 times, and 36 % more than five times. Parous women averaged five children (range 1–13). Approximately 25 % of women were pregnant and 23 % anaemic. *Plasmodium falciparum* infection accounted for 37 % (52/141) of malaria cases, 25 % (13/52) of which were assessed as severe. Among pregnant women, 11 % (31/286) had malaria infection compared to 15 % (110/755) of non-pregnant women. Among pregnant women with malaria, 35 % (11/31) were infected with *P. falciparum*, of which one was severe.

**Table 2:** Associations between demographic and clinical exposures and malaria, among 1150 case–control study participants in eastern Afghanistan

Variables	Cases, n (%)	Controls, n (%)	OR (95 % CI)	AOR (95 % CI)
	(N = 141)	(N = 1010)		
Age group				
15–20	32 (22.7)	286 (28.3)	Ref.	Ref.
21–30	62 (44.0)	437 (43.3)	1.27 (0.81–1.99)	1.36 (0.74–2.49)
31–49	47 (33.3)	287 (28.4)	1.46 (0.91–2.36)	1.49 (0.75–2.97)
Pregnant	31 (22.0)	255 (25.3)	0.83 (0.55–1.27)	0.89 (0.56–1.42)
Parity				
Nulliparous (no births)	36 (25.5)	256 (25.4)	Ref.	Ref.
Parous (1–5 births)	55 (39.0)	389 (38.5)	1.01 (0.64–1.57)	0.84 (0.47–1.52)
Grand-multiparous (6+ births)	50 (35.5)	365 (36.1)	0.97 (0.62–1.54)	0.68 (0.35–1.31)
Anaemic	33 (23.4)	230 (22.8)	1.04 (0.68–1.57)	1.00 (0.65–1.54)
ITN usage	45 (32.0)	431 (42.7)	0.63 (0.43–0.92)*	0.60 (0.40–0.91)*

* p < 0.05; AOR adjusted for district, facility, age, parity

Reported ITN usage was 32 % (45/141) among cases and 43 % (431/1010) among controls, giving a protective 40 % lower odds of malaria infection (AOR 0.60; 95 % CI 0.40–0.91). None of age, pregnancy status, parity, or anaemia were associated with malaria infection in univariate or multivariate analyses (Table 2).

### Community survey

#### Socioeconomic and clinical variables

In total, 530 pregnant women participated. Mean age was 28 (range 15–45) and 80 % had no formal education. Most households averaged 10 members (range 1–39), three under age five (range 0–18), living in three rooms (range 1–12). Most had no electricity (73 %; 387/530), while 48 % owned land (255/530). Table 3 shows education, employment and household assets used for principle components analysis, disaggregated by socioeconomic quartile.

**Table 3:** Factors used in principle components analysis to define socioeconomic quartiles among 530 community survey participants in eastern Afghanistan

Socioeconomic variables	Socioeconomic quartile, n (%)			
	1. Poorest (N = 133)	2. Poor (N = 132)	3. Less poor (N = 133)	4. Least poor (N = 132)
Education				
None	128 (30.2)	109 (25.8)	100 (23.6)	87 (20.5)
Religious/informal	3 (5.4)	15 (26.8)	18 (32.1)	20 (35.8)
Primary-school	2 (9.1)	6 (27.3)	3 (13.6)	11 (50.0)
Middle-school	0 (0)	1 (8.3)	4 (33.3)	7 (58.3)
High-school	0 (0)	1 (6.7)	8 (53.3)	6 (40.0)
University/technical	0 (0)	0 (0)	0 (0)	1 (100)
Primary earner’s employment				
Not working	2 (40.0)	2 (40.0)	1 (20.0)	0 (0)
Manual labour	70 (37.6)	46 (24.7)	43 (23.1)	27 (14.5)
Farming	35 (22.0)	48 (30.2)	34 (21.4)	42 (26.4)
Trade/market	14 (18.0)	22 (28.2)	20 (25.6)	22 (28.2)
Driver	8 (18.1)	10 (22.7)	11 (25.0)	15 (34.1)
Office/similar	4 (7.0)	4 (7.0)	24 (41.4)	26 (44.9)
Household assets				
Guestroom	28 (9.3)	70 (23.3)	91 (30.2)	112 (37.2)
Electricity	11 (7.4)	29 (19.7)	43 (29.3)	64 (43.5)
Land ownership	33 (13.0)	64 (25.1)	61 (24.0)	97 (38.0)
Car/truck	0 (0)	2 (4.0)	12 (24.0)	36 (72.0)
Radio/music-player	5 (4.2)	15 (12.7)	30 (25.4)	68 (57.6)
Rug	12 (6.6)	33 (18.2)	60 (33.2)	76 (42.0)
Curtains	18 (7.1)	57 (22.4)	71 (28.0)	108 (42.5)
Bicycle	25 (12.1)	38 (18.4)	60 (29.0)	84 (40.6)
Pressure-cooker	27 (8.3)	79 (24.2)	101 (31.0)	119 (36.5)
ITNs	9 (4.5)	31 (15.6)	65 (32.7)	94 (47.2)

Malaria point prevalence was 3.8/1000 (95 % CI 0.9–15.0), anaemia was 277/1000 (95 % CI 241–317), and severe anaemia 34/1000 (95 % CI 21–53), similar to delivery-ward survey findings. As only two malaria cases were detected, analysis of effects on maternal haemoglobin concentration was conducted for anaemia instead. Table 4 shows none of age, parity, trimester, malaria infection, iron/folate usage, antenatal attendance, ITN usage, or SES were associated with anaemia in multivariate analysis.

**Table 4:** Associations of socioeconomic, clinical and behavioural responses with anaemia among 530 community survey participants in eastern Afghanistan

Variables	Hb	Anaemic, n (%)	Non-anaemic, n (%)	OR (95 % CI)	AOR (95 % CI)
	Mean ± SD (range)	(N = 147)	(N = 383)		
Age					
15–20	11.4 ± 1.9 (7.3–19.3)	22 (15.0)	78 (20.4)	Ref.	Ref.
21–30	11.0 ± 1.7 (6.5–17.5)	81 (55.1)	223 (58.2)	1.29 (0.75–2.20)	1.35 (0.71–2.57)
31–45	10.7 ± 1.7 (6.5–15.5)	44 (29.9)	82 (21.4)	1.90 (1.05–3.46)*	1.89 (0.85–4.20)
Parity					
Nulli/primiparous (0–1 births)	11.1 ± 1.8 (6.8–17.5)	35 (23.8)	103 (27.0)	Ref.	Ref.
Multiparous (2–5 births)	10.9 ± 1.7 (6.5–19.3)	76 (51.7)	187 (48.8)	1.20 (0.75–1.90)	0.99 (0.57–1.72)
Grand-multiparous (6+ births)	10.9 ± 1.7 (6.5–14.7)	36 (24.5)	93 (24.3)	1.14 (0.67–1.96)	0.76 (0.38–1.52)
Trimester					
1st	10.9 ± 2.1 (8.0–14.0)	3 (2.0)	6 (1.58)	Ref.	Ref.
2nd	11.3 ± 1.7 (7.0–17.5)	57 (38.8)	188 (49.1)	0.61 (0.15–2.50)	0.53 (0.12–2.41)
3rd	10.7 ± 1.7 (6.5–19.3)	87 (59.2)	189 (49.4)	0.92 (0.22–3.77)	0.76 (0.17–3.41)
Malaria infection					
No	11.0 ± 1.7 (6.5–19.3)	147 (100)	381 (99.5)	–	–
Yes	12.5 ± 1.4 (11.5–14)	0 (0)	2 (0.5)	–	–
Iron/folate usage					
No	10.9 ± 1.7 (6.5–19.3)	108 (73.5)	277 (72.3)	Ref.	Ref.
Yes	11.1 ± 1.7 (6.7–15.5)	39 (26.5)	106 (27.7)	0.94 (0.61–1.44)	1.16 (0.73–1.84)
Antenatal attendance (at least once)					
No	11.0 ± 1.4 (6.9–17.5)	20 (13.6)	54 (14.1)	Ref.	Ref.
Yes	11.0 ± 1.7 (6.5–19.3)	127 (86.4)	329 (85.9)	1.04 (0.60–1.81)	1.03 (0.57–1.87)
Household ITN ownership					
No	11.0 ± 1.8 (6.5–19.3)	91 (61.9)	240 (62.7)	Ref.	Ref.
Yes	11.1 ± 1.7 (6.5–15.5)	56 (38.1)	143 (37.3)	1.03 (0.70–1.53)	1.74 (0.99–2.90)
Slept under ITN last night					
No	11.0 ± 1.4 (6.5–19.3)	134 (91.2)	357 (93.2)	Ref.	Ref.
Yes	10.8 ± 1.4 (8.6–14.2)	13 (8.8)	26 (6.8)	1.33 (0.66–2.67)	1.71 (0.82–3.64)
Socioeconomic status					
1. Poorest	10.8 ± 1.9 (6.5–17.5)	47 (32.0)	86 (22.5)	Ref.	Ref.
2. Poor	10.9 ± 1.8 (6.8–19.3)	35 (23.8)	97 (25.3)	0.66 (0.39–1.12)	0.72 (0.41–1.26)
3. Less poor	10.9 ± 1.7 (6.5–14.3)	39 (26.5)	94 (24.5)	0.76 (0.45–1.27)	0.95 (0.53–1.71)
4. Least poor	11.4 ± 1.6 (7.5–15.5)	26 (17.7)	106 (27.7)	0.45 (0.26–0.78)*	0.57 (0.29–1.10)

* p < 0.05; AOR adjusted for survey, age, parity, trimester, SES, district; Cell sizes below 30 use exact logistic methods

#### Knowledge/perceptions

Malaria knowledge was high, with 99 % reporting fever, shivering/chills, headache, weakness or joint pain as symptoms, 97 % reporting mosquito bites transmit malaria, 70 % reporting diagnosis by blood test, and 81 % reporting ITN usage as the best available prevention. Risk perception was also high, with 85 % proposing malaria as their community’s ‘worst health problem,’ 90 % as common in their community, and 38 % reporting they had experienced ‘malaria’ during their present pregnancy. In contrast, only 6 % identified either diarrhoeal disease or acute respiratory tract infections as concerns, despite high frequency of both (Table 5).

**Table 5:** Associations of knowledge and behavioural responses with socioeconomic status among 530 community survey participants in eastern Afghanistan

Response variables	Poorer^a^	Wealthier^a^	OR (95 % CI)	AOR (95 % CI)
	(N = 265)	(N = 265)		
Primary source of healthcare				
NGO/government health facility	217 (81.9)	184 (69.4)	Ref.	Ref.
Private health facility	43 (16.2)	67 (25.3)	1.84 (1.19–2.82)*	1.30 (0.77–2.21)
Traditional/self-treat	5 (1.9)	14 (5.3)	–	–
Attended antenatal services at least once	228 (86.0)	228 (86.0)	1.00 (0.61–1.63)	1.38 (0.75–2.54)
Uses iron/folate supplements	54 (20.45)	91 (34.3)	2.34 (1.55–3.53)*	1.90 (1.17–3.09)*
Greatest health concern				
Other	3 (1.1)	10 (3.8)	–	–
Diarrhoea	18 (6.8)	14 (5.3)	Ref.	Ref.
ARI	12 (4.5)	21 (7.9)	0.53 (0.12–2.29)	0.53 (0.09–3.03)
Malaria	232 (87.6)	220 (83.0)	0.28 (0.77–1.05)	0.43 (0.91–2.00)
How common is malaria				
No malaria/infrequent	22 (8.3)	29 (10.9)	Ref.	Ref.
Common	243 (91.7)	236 (89.1)	0.74 (0.41–1.32)	0.73 (0.36–1.48)
Best malaria prevention in pregnancy				
Nothing works	29 (10.9)	24 (9.1)	Ref.	Ref.
ITNs	212 (80.0)	219 (82.6)	1.25 (0.70–2.21)	1.26 (0.64–2.46)
Rapid diagnosis/treatment	1 (0.4)	1 (0.4)	–	–
Burning/smoke	8 (3.0)	9 (3.4)	–	–
Clean house/area	15 (5.7)	12 (4.5)	0.97 (0.38–2.46)	0.53 (0.17–1.64)
Preferred malaria diagnosis				
Self/informal	31 (11.7)	48 (18.1)	Ref.	Ref.
Facility (clinical)	50 (18.9)	29 (10.9)	0.37 (0.20–0.71)*	0.43 (0.19–0.96)*
Facility (blood test)	184 (69.4)	188 (70.9)	0.66 (0.40–1.08)	0.91 (0.48–1.72)
Preferred malaria treatment				
NGO/government health facility	214 (80.8)	198 (74.2)	Ref.	Ref.
Private health facility/Other	51 (19.2)	67 (25.3)	1.42 (0.94–2.14)	0.86 (0.51–1.44)
Would use malaria-preventive drugs in pregnancy				
Never	232 (87.6)	204 (77.0)	Ref.	Ref.
Yes/maybe	33 (12.5)	61 (23.0)	2.10 (1.32–3.34)*	1.69 (0.98–2.91)
Why use ITNs				
Avoid mosquitoes	169 (63.8)	167 (63.0)	Ref.	Ref.
Prevent insect bites	49 (18.5)	43 (16.2)	0.89 (0.56–1.41)	1.15 (0.65–2.02)
Prevent malaria	40 (15.1)	51 (19.3)	1.29 (0.81–2.06)	1.92 (1.07–3.43)*
Other/don’t know	7 (2.6)	4 (1.5)	–	–

ITN-owners only	n = 40 (%)	n = 159 (%)		
Which family members use ITNs				
Nobody/unknown	11 (27.5)	41 (25.8)	Ref.	Ref.
All	9 (22.5)	61 (38.4)	1.82 (0.69–4.78)	1.23 (0.37–4.04)
Children	20 (50.0)	43 (27.0)	0.58 (0.24–1.35)	0.48 (0.16–1.40)
Women	0 (0)	11 (6.9)	–	–
Men/elderly	0 (0)	3 (1.9)	–	–
Participant used ITN last night	7 (17.5)	32 (20.1)	1.19 (0.48–2.93)	3.09 (1.01–9.51)*

Reasons for not using ITN last night	n = 33 (%)	n = 127 (%)		
No mosquito nuisance	11 (33.3)	35 (27.6)	Ref.	Ref.
ITN used by others	12 (36.4)	26 (20.5)	0.68 (0.26–1.78)	2.87 (0.42–19.8)
No reason provided	10 (30.3)	66 (52.0)	2.07 (0.80–5.36)	4.65 (1.09–19.8)*

* p < 0.05; AOR adjusted for survey, age, parity, trimester, district

^a^Poorer merges SEQ 1 and 2, Wealthier merges SEQ 3 and 4

#### Reported practices

Most (76 %) reported NGO-run public-sector facilities as their primary source of healthcare. Most (86 %) reported attending antenatal services at least once during pregnancy, though only 42 % (223/530) reported attending 3–4 times as recommended by national guidelines. Only 27 % reported taking iron/folate during pregnancy, with 55 % reporting no use of dietary supplements. However, this differed by SES, with wealthier women having almost double the odds of taking iron/folate (AOR 1.90; 95 % CI 1.17–3.09).

Most (78 %) reported using public facilities for malaria treatment, primarily due to affordability (45 %) and effectiveness (40 %), with no differences by SES. Most (82 %) reported they would never take drugs to prevent malaria during pregnancy. Of almost half (43 %) reporting avoiding all medicines during pregnancy, 55 % reported doing so because they might feel sick and 32 % because drugs—including anti-malarials—might be dangerous during pregnancy. Although most (81 %) recommended ITNs for malaria prevention, only 17 % reported this as their major advantage and most (81 %) identified avoiding nuisance biting as most important. Approximately 38 % reported household ownership of at least one ITN. Most women in ITN-owning households reported that everyone (35 %) or children (32 %) usually slept under ITNs. While 80 % reported not using an ITN the previous night, wealthier women had three times higher odds of having slept under one (AOR 3.09; 95 % CI 1.01–9.51).

## Discussion

### Prevalence and perceptions

This study design, by using three distinct data sources to examine the scope of the problem in Afghanistan, provides a broad view of malaria and anaemia in pregnancy. It is clear that factors other than malaria are chiefly responsible for the high prevalence of maternal anaemia detected in delivery-ward and cross-sectional surveys. Malaria prevalence of only 3.9/1000 among pregnant women in communities and 1.9/1000 in a delivery-ward, while still warranting concern, appears to reflect a genuine reduction in malaria transmission rates in Afghanistan [31]. While not directly comparable, community survey data indicates malaria prevalence among all age groups in eastern Afghanistan has fallen from typically recorded prevalence of 7–10 % for vivax and 2–5 % for falciparum malaria in 2000 to approximately 0.16 % for vivax and 0.01 % for falciparum in 2013 [33, 40]. Reasons for this apparent reduction could include expansion of health services [23, 30], increased availability of malaria diagnosis and treatment [41], enhanced control activities [40, 42], improved political stability and socioeconomic development [30], and/or changing environmental conditions and improved agricultural practices [28].

While recorded malaria prevalence was low, the high prevalence of maternal anaemia—consistent with a reported national prevalence of 40.3 % among WRA—is a concern [43]. However, associations between anaemia and malaria were not observed. More likely contributors were poor diet, lack of access to nutritional supplements during pregnancy, and poor spacing and high frequency of pregnancies [40]. Intestinal parasite prevalence was higher than the 3.8 % reported among pregnant women in the 2013 National Nutrition Survey [43]. LBW prevalence was lower than the Unicef estimate of 28 % for the South Asia region, though comparable data was unavailable for Afghanistan [44].

Perceptions of malaria risk appeared higher than warranted by a prevalence of 1.9–3.8/1000. It is unlikely that most of the 38 % reported malaria in the community survey was actually malaria, and it was not associated with anaemia prevalence. Both Leslie et al. and Reynolds et al. found febrile illness was often erroneously clinically diagnosed as malaria, while Howard et al. found that malaria and typhoid were often identified interchangeably in communities [41, 45, 46]. It should also be noted that women were aware that the survey focused on malaria, and reporting it as a ‘major health concern’ could have reflected both perceived risk and some potential response bias. Perceptions perhaps predated health system strengthening and/or were encouraged by popular misconceptions that non-specific febrile illness was often malarial [41, 45]. Despite minimal educational attainment, women were knowledgeable about malaria, probably reflecting long-running health education efforts by NGOs since the 1990s [46]. Growing evidence indicates messages have been effective, despite low literacy and cultural constraints targeting most efforts at men [32, 46].

### Policy and practice implications

This study demonstrated that the risk of malaria in pregnancy is low among Afghan women. However, the malaria burden among pregnant women in Afghanistan remains sufficient to warrant specific action. *Plasmodium vivax* treatment is particularly challenging in pregnancy. While blood-borne parasites respond to chloroquine or sulfadoxine–pyrimethamine, both safe in pregnancy [47], vivax infections often relapse without radical primaquine treatment [48]. Pregnant and lactating women cannot receive primaquine, due to risks of haemolytic anaemia in glucose-6-phosphate dehydrogenase (G6PD) deficient foetuses [22]. Pregnant women thus risk repeated clinical episodes throughout pregnancy and lactation, as well as pre-term, miscarried, and LBW deliveries [1, 4, 6, 10–15, 19, 20]. Given the challenges of infection in pregnancy, prevention is clearly preferable. The two prevention approaches advocated by WHO in pregnancy are (1) ITN/LLINs, and (2) intermittent preventive treatment in pregnancy (IPTp), providing a full therapeutic anti-malarial course during antenatal visits.

Which approach would be most effective and acceptable for Afghanistan? ITN/LLINs demonstrated 40 % malaria protection in the case–control study, similar to protection demonstrated for Afghan refugee populations in neighbouring Pakistan [49]. Additionally, the community survey confirmed ITN/LLINs were popular and used by all family members [32, 33]. Conversely, persuading pregnant women to take IPTp could be challenging given the perceived risks of taking medication during pregnancy. Weighing the limited risk of malaria in pregnancy, demonstrable protection and popularity of LLINs, reported avoidance of drugs during pregnancy, and still relatively limited data on IPTp for vivax malaria, there seems no real justification to initiate an IPTp strategy in Afghanistan. ITNs/LLINs are a proven preventive strategy, which along with rapid diagnosis and treatment, can help protect pregnant women in low-endemicity countries such as Afghanistan [33]. Universal coverage with ITNs/LLINs, such as the Global Fund supported initiative providing free LLINs to pregnant women and immunized children, should be the preventive strategy of choice for pregnant women in Afghanistan.

Less than half of community survey participants reported household ownership of any ITNs and only 20 % reported using ITNs the previous night, irrespective of season or socioeconomic status. Since this study universal LLIN coverage campaigns have increased coverage to approximately 80 % of high-risk populations [40]. LLINs can be used for an average 3–5 year lifespan without retreatment, and since 2005 WHO has recommended programmes only purchase and distribute LLINs [50]. Health messages should emphasize that LLINs be used by all family members, particularly those at greatest risk from malaria. That over 80 % of women cited prevention of nuisance biting as more relevant than malaria prevention is not necessarily negative, as families do use ITNs appropriately when given access [31].

Despite the benefits of ITNs, they may not be sufficient to reduce malaria in pregnancy on their own, as 32 % of malaria cases in the case–control study used ITNs. Intermittent screening and treatment in pregnancy (ISTp), during routine antenatal visits, has shown some promise in other settings and could be considered [51–54]. However, as malaria is normally symptomatic in Afghanistan and women seek treatment promptly, the benefits of adding ISTp to antenatal services would likely be relatively small even with increased antenatal attendance. National antenatal guidelines currently recommend that women in malarious areas be advised on prompt treatment seeking and usage of ITNs [55]. Thus, policy recommendations on ISTp would only be possible after research on acceptability, costs, and effectiveness.

### Limitations

Low malaria prevalence limited analysis of associations. Reliance on prevalence data during pregnancy can underestimate malaria incidence risk in low transmission areas, as described by Rijken et al., and longitudinal calculation of cumulative incidence would have been preferable [6]. Additionally, community sampling may have underestimated malaria prevalence if those ill at home did not participate. However, comparison of facility and community samples produced no evidence of bias, indicating surveys reflected transmission. Use of febrile outpatient rather than community controls is a limitation, as controls may have been previously parasitaemic. A population control, though preferable, was not feasible.

## Conclusions

Malaria did not appear responsible for the high prevalence of maternal anaemia detected. While malaria prevalence was low, the risk of severe malaria among pregnant women is sufficient in Afghanistan to justify specific preventive interventions. Given women’s perceptions of drug usage in pregnancy and the limited transmission risk, an IPTp implementation strategy is not justified. Scaling-up LLINs, with increased MOPH emphasis on usage in pregnancy, is likely to be more successful in Afghanistan.
